# Knowledge and Perception of Caregivers Regarding Adverse Events Following Immunization and Associated Predictors at Hospitals in Osogbo, Southwestern Nigeria: A Cross‐Sectional Study

**DOI:** 10.1002/hsr2.73058

**Published:** 2026-08-26

**Authors:** Funso Abidemi Olagunju, Samuel Olorunyomi Oninla, Sunday Charles Adeyemo, Abimbola Ololade Odeyemi, Kehinde Awodele, Efeturi Agelebe, Abisola Oluwatoyin Omoboyeje, Muritala Adewale Ibrahim, Ayodeji Oluwaseun Ogungbemi, Ayodele Raphael Ajayi, Eniola Dorcas Olabode, Oladunni Opeyemi, Olusola Adetunji Oyedeji

**Affiliations:** ^1^ Department of Paediatrics and Child Health Osun State University Osogbo Nigeria; ^2^ Department of Paediatrics and Child Health Ladoke Akintola University of Technology Ogbomoso Nigeria; ^3^ Institut Superieur de Sante Niamey Niger Republic; ^4^ Department of Obstetrics and Gynaecology Osun State University Teaching Hospital Osogbo Nigeria; ^5^ Department of Paediatrics and Child Health Bowen University Iwo Nigeria; ^6^ Department of Family Medicine Osun State University Osogbo Nigeria; ^7^ Department of Psychiatry Medicine Afe Babalola University Ado‐Ekiti Nigeria; ^8^ Public Health Department Adeleke University Ede Nigeria; ^9^ Department of Paediatrics and Child Health Osun State University Teaching Hospital Osogbo Nigeria

**Keywords:** adverse events following immunization, caregivers, immunization, knowledge, Nigeria, perception

## Abstract

**Background and Aims:**

Adverse events following immunization (AEFI) can erode trust in vaccines and contribute to immunization dropout. Caregivers usually observe an AEFI first and decide whether it is reported and whether the child returns for later doses, yet their knowledge and perception are rarely assessed together. This study assessed caregivers' knowledge and perception of AEFI, and associated factors, in Osogbo, Southwestern Nigeria.

**Methods:**

This hospital‐based cross‐sectional study included 368 caregivers presenting children for routine vaccination at three hospitals in Osogbo between October 15, 2025, and February 2, 2026, selected by proportionate systematic random sampling. A pretested, self‐administered questionnaire captured sociodemographic characteristics and 10 knowledge and 10 perception items; scores were graded poor (0–3), fair (4–6), or good (7–10). Associated factors were examined with Pearson's *χ* ^2^ test, independent predictors with multinomial logistic regression (good grade as reference), and correlation with Pearson's *r*. All tests were two‐sided; significance was set at *p* < 0.05 (SPSS version 27.0).

**Results:**

Most caregivers had good knowledge (254; 69.0%) and good perception (220; 59.8%), but only 216 (58.7%) knew an AEFI may occur without a causal link to the vaccine. Age group and residential status were associated with knowledge; these, plus educational and occupational status and socioeconomic class, were associated with perception (all *p* < 0.05). Caregivers aged 25–34 years (AOR 0.30, 95% CI 0.089–0.998) and those living within Osogbo (AOR 0.44, 95% CI 0.225–0.84) had lower odds of fair versus good knowledge. Knowledge and perception scores were moderately correlated (*r* = 0.442, *p *< 0.001).

**Conclusion:**

Knowledge and perception of AEFI were largely good, yet many caregivers attribute every post‐vaccination event to the vaccine. Interventions targeting caregivers with lower education, lower socioeconomic status and peri‐urban residence, delivered through immunization clinics, community leaders and social media, may improve AEFI literacy and reduce vaccine hesitancy.

**Abbreviations**
AEFI: adverse events following immunization
AOR: adjusted odds ratio
BCG: Bacille Calmette–Guerin
CI: confidence interval
HBV: hepatitis B vaccine
HCW: health care worker
OLFCH: our lady of fatima catholic hospital
OPV: oral polio vaccine
SD: standard deviation
SDG: sustainable development goals
SPSS: statistical package for the social sciences
SSHA: state specialist' hospital, Asubiaro
STROBE: strengthening the reporting of observational studies in epidemiology
UTH: UNIOSUN teaching hospital
VPD: vaccine‐preventable diseases

## 1 Introduction

It is not an overstatement that vaccination has contributed immensely to the prevention and control of infectious diseases, especially the vaccine‐preventable diseases, since the first successful vaccine was introduced by Dr. Edward Jenner in May 1798 [1]. Despite this great achievement in global health over the years, adverse events following immunization (AEFI) have been identified as one of the impediments that could lead to loss of public trust in vaccines and, in the long run, to vaccine hesitancy, that is, a delay in acceptance or refusal of vaccines despite the availability of vaccination services [2].

Adverse events following immunization are untoward medical occurrences that follow the administration of a vaccine or combination of vaccines, and which are not necessarily causally linked to the vaccine [3]. They remain a source of public health concern globally, and more importantly in the developing world, where vaccine illiteracy, poor healthcare funding, and inadequate healthcare personnel plague the healthcare system [4]. They can therefore impact negatively on the vital statistics of any nation [4].

Caregivers occupy a pivotal position along the AEFI pathway. They are usually the first to observe an event, to judge whether it warrants attention, to decide whether it is brought to the notice of a health worker, and ultimately to decide whether the child returns for subsequent doses. Their knowledge and perception therefore bear directly on the completeness of AEFI surveillance, on the timeliness of care for serious events, and on immunization dropout [5]. Where knowledge and perception are sub‐optimal, fear, misconception, rumor and conspiracy narratives fill the gap, and the outlook for the immunization program becomes perilous [6].

Varying levels of knowledge of AEFI have been demonstrated by caregivers across Nigeria. Afolaranmi et al. [6] in a study in Jos, North Central Nigeria, reported that 31.5% of caregivers had good knowledge and 68.5% poor knowledge of AEFI. In another related study, Issa‐Onilu et al. [7] reported 78.9% adequate knowledge and 21.1% inadequate knowledge of AEFI in Ilorin, Nigeria. Several other studies have assessed knowledge and perception of AEFI among various cadres of healthcare workers [5, 8].

Three gaps nevertheless remain. First, knowledge and perception have rarely been measured in the same caregiver population, so the extent to which one tracks the other, and therefore whether interventions that raise knowledge can be expected to shift perception, is not established. Second, most caregiver studies have stopped at bivariate description and have not identified independent, potentially modifiable correlates after adjustment. Third, no published data describe caregivers in Osun State, a Southwestern setting whose sociocultural context and immunization service delivery differ from the northern settings previously studied. These gaps are not merely descriptive: identifying which caregiver groups are least well informed indicates where health education, community engagement, and social media messaging should be concentrated if AEFI reporting is to improve and immunization dropout is to fall.

This study, therefore, set out to assess the knowledge and perception of caregivers regarding adverse events following immunization, and the associated factors, at hospitals in Osogbo, Southwestern Nigeria, so as to identify gaps and inform targeted interventions.

## 2 Methods

### 2.1 Study Design and Reporting

This was a hospital‐based, descriptive cross‐sectional study conducted over 4 months, from October 2025 to February 2026. The study is reported in accordance with the STROBE (Strengthening the Reporting of Observational Studies in Epidemiology) statement for cross‐sectional studies. Statistical reporting follows the SAMPL (Statistical Analyses and Methods in the Published Literature) guidelines.

### 2.2 Study Setting

The study was conducted at Osun State University (UNIOSUN) Teaching Hospital, State Specialists' Hospital Asubiaro, and Our Lady of Fatima Catholic Hospital, all in Osogbo, the capital of Osun State.

UNIOSUN Teaching Hospital (UTH) is a tertiary hospital with 350 beds. It offers specialized and general health care to the inhabitants of Osogbo and its environs. The study was carried out at the immunization center of the hospital. An average of 12,000 children are vaccinated at this center annually. It is open every working day between the hours of 8 a.m. and 4 p.m. The center is under the supervision of a Deputy Director of Nursing, assisted by other nurses and community health extension workers. The services rendered at the center include vaccination of newborns, infants, older children, and adults. Others are health education and promotion on topics such as immunization, breastfeeding and infant nutrition, and family planning for mothers. Follow‐up care of vaccinated children who have complaints arising from vaccination is also ensured, and if need be, doctors attached to the pediatric unit evaluate and manage such complaints.

The State Specialist Hospital, Asubiaro (SSHA) is a 200‐bed hospital which offers secondary‐level health care. It is a referral center for most of the primary health care centers and comprehensive health centers in the State. An average of 24,000 children are vaccinated at the facility annually. Its immunization unit opens every day. The unit is overseen by a Deputy Director of Nursing, assisted by other nurses and community health extension workers. The services rendered at the unit are similar to those of the teaching hospital.

Our Lady of Fatima Catholic Hospital (OLFCH) is a missionary hospital established in 1957. It is an 80‐bed hospital and also offers secondary‐level health care. Its immunization unit runs every Tuesday, with an average of 2400 children vaccinated annually.

### 2.3 Study Population

The study population comprised caregivers who presented their infants or children for vaccination at the immunization centers of the three hospitals. The target population was caregivers whose infants or children had received at least the first routine vaccination schedule, that is, Bacille Calmette–Guerin (BCG), oral polio vaccine (OPV), and hepatitis B vaccine (HBV).

### 2.4 Inclusion and Exclusion Criteria

#### 2.4.1 Inclusion Criteria

Caregivers whose infants or children had received at least the first routine vaccination schedule (BCG, OPV, and HBV) and who gave written informed consent to participate.

#### 2.4.2 Exclusion Criteria

Caregivers who were critically ill, mentally impaired, or had impaired memory.

### 2.5 Sample Size Determination

The sample size was determined using the Leslie Fisher's formula [9]:

$${n=Z}^{2}{\text{pq}/d}^{2}$$

where *n *= minimum sample size; *Z *= standard normal deviate at the 95% confidence level (1.96); *p *= proportion in the characteristic being measured (*p* = 68.5%, the proportion with poor knowledge reported by Afolaranmi et al. [6]); *q* = 1 − p; and *d *= precision, set at 0.05.

$$n=1.96^{2}\times 0.685\times 0.315/0.05^{2}=331.56$$

The minimum sample size was therefore approximately 332. A 10% allowance was added for possible non‐response and attrition, giving a target of 400 participants.

### 2.6 Sampling Technique

The number of participants recruited from each facility was determined by proportionate allocation, using the ratio of the average annual vaccination uptake of each facility to the total uptake of the three facilities. This ratio was approximately 1:5:10 for OLFCH, UTH, and SSHA, respectively, amounting to 23, 115, and 230 participants for OLFCH, UTH, and SSHA, respectively.

Participants were then selected within each facility by systematic random sampling. The sampling frame for each facility was the daily immunization attendance register maintained by the immunization unit staff, which was obtained from the unit at the start of each immunization session; it was not compiled by observation on the part of the research team. Because data collection ran across the full 4‐month study period rather than on a single day, and because daily client volume varies, a single sampling interval (*k*) per facility was computed from the average session attendance recorded in that facility's register in relation to the number of participants allocated to the facility, rather than being recalculated on each data‐collection day. The resulting intervals were *k* = 16 for OLFCH, *k* = 3 for UTH, and *k* = 2 for SSHA. On each data‐collection day, the first participant was selected by balloting among the first *k* caregivers listed on the register, and every *k*th caregiver thereafter was approached, until the allocated quota for the facility was reached.

### 2.7 Study Instrument and Scoring

A self‐administered, semi‐structured questionnaire was adapted from previous studies [6, 7] and modified for contextual applicability. It was pretested at the Immunization Center of Cottage Hospital, Ede, Osun State, using 10% of the sample size (*n* = 37); pretest participants were not included in the final analysis. Content validity was ensured by expert review by consultant public health physicians, and internal consistency was acceptable (Cronbach's alpha = 0.8). The questionnaire consisted of three sections.

The first section captured the sociodemographic characteristics of the participants. Socioeconomic class was determined using the Ibadin and Akpede scoring scheme [10].

The second section contained 10 questions assessing knowledge of AEFI. Each correct answer scored one mark, giving a maximum of 10 marks. Knowledge was graded as poor (0–3), fair (4–6), or good (7–10).

The third section contained 10 questions assessing perception of AEFI. Each correct or favorable response scored one mark, giving a maximum of 10 marks. Perception was graded as poor (0–3), fair (4–6), or good (7–10).

### 2.8 Statistical Analysis

Completed questionnaires were checked manually for completeness and internal consistency, coded, and analyzed using IBM SPSS Statistics version 27.0 (IBM Corp., Armonk, NY, USA). Categorical variables were summarized as frequencies and percentages, and continuous variables as means with standard deviations (SD).

Two outcomes were pre‐specified: knowledge of AEFI and perception of AEFI, each graded as poor, fair or good as described above. The association between each outcome and the sociodemographic variables (age group, sex, relationship to the vaccinated child, residential status, educational status, occupational status and socioeconomic class) was examined using Pearson's *χ* ^2^ test, with the test statistic (*χ* ^2^), degrees of freedom (df), and *p* value reported alongside the underlying cell frequencies. *χ* ^2^ testing establishes association only, not prediction, and these analyses were therefore treated as a screening step. Variables significant at *p* < 0.05 in this step were entered into multinomial logistic regression models to identify independent predictors, with the “good” grade as the reference outcome category. Adjusted odds ratios (AOR) are reported with 95% confidence intervals (CI).

Although knowledge and perception are ordered categories, multinomial rather than ordinal (proportional‐odds) logistic regression was used, because the multinomial model does not impose the proportional‐odds assumption and permits the “fair” and “poor” grades to be contrasted separately against “good”. Estimates are accordingly interpreted as category‐specific contrasts and not as a single ordinal effect.

The relationship between knowledge and perception scores was assessed as a pre‐specified secondary outcome using Pearson's correlation coefficient (*r*), interpreted as weak (*r* < 0.30), moderate (*r* = 0.30–0.59), or strong (*r* ≥ 0.60). All item‐level comparisons are exploratory and are reported as such.

All tests were two‐sided and the a priori level of statistical significance was *p* < 0.05. *p* values are reported as *p* < 0.001 for values below 0.001, to three decimal places for values between 0.001 and 0.01, and to two decimal places for values of 0.01 or greater. *p* values are presented together with the underlying frequencies, effect estimates, and the test used to derive them.

### 2.9 Ethical Approval and Consent

The study was conducted in accordance with the ethical principles of the Declaration of Helsinki. Ethical approval was obtained from the Osun State Health Research Ethics Committee (protocol number OSHREC/PRS/569T/1279). Permission was also obtained from the management of each participating hospital. Written informed consent was obtained from every participant before enrollment, after the purpose, procedures, voluntary nature, risks, and benefits of the study had been explained to them. Participation was voluntary, participants were free to withdraw at any point without any effect on the services their children received, and questionnaires were anonymised and stored securely, accessible only to the research team. All ethical principles guiding the conduct of research, including beneficence, non‐maleficence, confidentiality, justice, and respect for autonomy, were strictly adhered to.

## 3 Results

Of the 400 caregivers recruited, 368 returned correctly and completely filled questionnaires, giving a response rate of 92.0%.

### 3.1 Sociodemographic Characteristics of the Participants

Nearly all participants were female (365; 99.2%), and only 3 (0.8%) were male. Mothers constituted the largest proportion (358; 97.3%), followed by aunts (7; 1.9%) and fathers (3; 0.8%). Most participants (313; 85.1%) resided within Osogbo, while 55 (14.9%) lived outside Osogbo. The mean age of the participants was 30.0 ± 4.8 years. Other details are shown in Table 1.

**Table 1:** Sociodemographic characteristics of the participants (*N* = 368).

Variable	Frequency (*n*)	Percentage (%)
Sex		
Female	365	99.2
Male	3	0.8
Age group (years)		
15–24	18	4.9
25–34	200	54.3
35–44	143	38.9
45–54	7	1.9
Relationship to the vaccinated child		
Mother	358	97.3
Aunt	7	1.9
Father	3	0.8
Residential status		
Within Osogbo	313	85.1
Outside Osogbo	55	14.9
Educational status		
Primary	38	10.3
Secondary	176	47.8
Tertiary	149	40.5
Informal	5	1.4
Occupational status		
Civil servant	88	23.9
Artisan	73	19.8
Trading	192	52.2
Farming	1	0.3
Unemployed	14	3.8
Socioeconomic class		
Low	148	40.2
Middle	158	42.9
Upper/high	62	16.9

*Note:* Percentages are of the total sample (*N* = 368) and may not sum to exactly 100 because of rounding.

### 3.2 Knowledge of Participants Regarding Adverse Events Following Immunization

**Figure 1:**
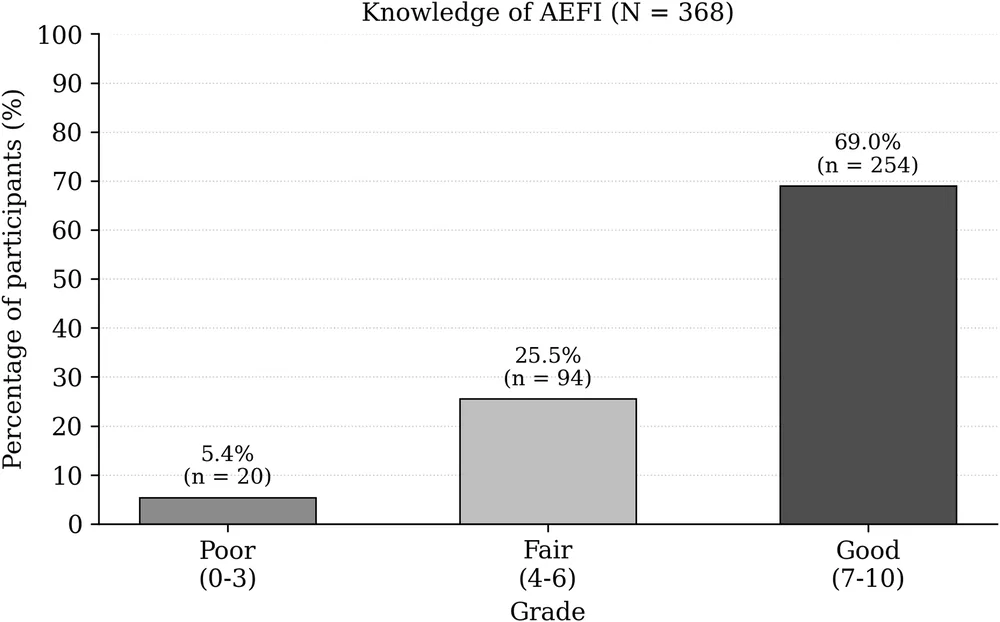
Distribution of participants' knowledge scores regarding AEFI (*N* = 368). Knowledge was categorized into three grades: Poor (0–3), Fair (4–6), and Good (7–10). Data are presented as percentages with absolute frequencies (*n*) shown in parentheses above each bar. The majority of participants (69.0%, *n* = 254) demonstrated a “Good” level of knowledge.

Responses to the knowledge items are shown in Table 2. The item answered correctly by the largest proportion of participants was “Do you know caregivers need to watch out for AEFI?” (KQ9), answered correctly by 314 (85.3%). The item answered correctly by the smallest proportion was “Do you know AEFI can occur without any link to the vaccine?” (KQ5), answered correctly by 216 (58.7%). Overall, 20 participants (5.4%) had poor knowledge, 94 (25.5%) fair knowledge, and 254 (69.0%) good knowledge (Figure 1). Notably, the “good” category included participants who missed conceptually critical items such as KQ5.

**Table 2:** Participants' responses to the knowledge items on AEFI (*N* = 368).

Knowledge item	Yes, *n* (%)	No, *n* (%)
KQ1. Are you aware some medical complaints may occur following vaccination?	291 (79.1)	77 (20.9)
KQ2. Do you know AEFI may be due to inappropriate handling of the vaccines?	267 (72.6)	101 (27.4)
KQ3. Do you know AEFI can present with serious and non‐serious ailments?	293 (79.6)	75 (20.4)
KQ4. Are you aware AEFI may present with high‐grade fever?	300 (81.5)	68 (18.5)
KQ5. Do you know AEFI can occur without any link to the vaccine?	216 (58.7)	152 (41.3)
KQ6. Do you know background medical conditions predispose to AEFI?	238 (64.7)	130 (35.3)
KQ7. Do you know AEFI can lead to loss of confidence in vaccination programs?	290 (78.8)	78 (21.2)
KQ8. Do you know health education about AEFI is needed before commencement of vaccination?	274 (74.5)	94 (25.5)
KQ9. Do you know caregivers need to watch out for AEFI?	314 (85.3)	54 (14.7)
KQ10. Do you know the benefits of immunization outweigh AEFI?	283 (76.9)	85 (23.1)

Abbreviations: AEFI = adverse events following immunization, KQ = knowledge question.

### 3.3 Perception of Participants Regarding Adverse Events Following Immunization

**Figure 2:**
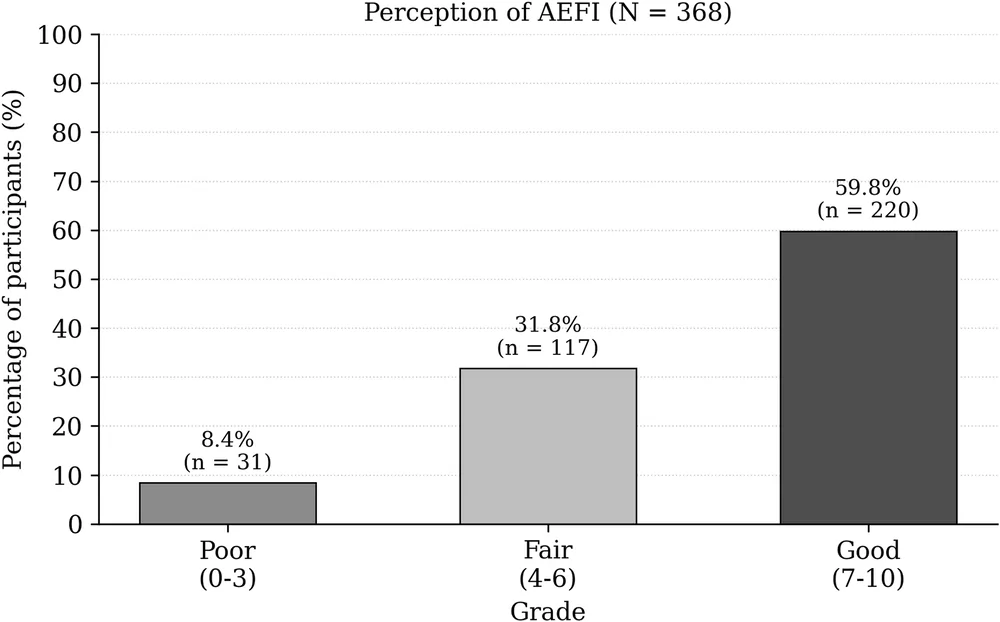
Distribution of participants' perception scores regarding AEFI (*N* = 368). Perception levels were stratified into three grades: poor (0–3), fair (4–6), and good (7–10). The bars indicate the percentage of participants in each grade, with exact proportions and absolute frequencies (*n*) displayed above the bars.

Responses to the perception items are shown in Table 3. Two‐thirds of participants (245; 66.6%) responded “Yes” to “Do you think there is nothing to worry about AEFI?” (PQ1). Sixty‐two participants (16.8%) responded “Yes” to “If your child has AEFI, would that stop you from receiving other vaccines?” (PQ5), while the majority (306; 83.2%) responded “No”. One hundred and fifty‐one participants (41.0%) believed that health workers would blame them for reporting an AEFI (PQ7). Overall, 31 participants (8.4%) had poor perception, 117 (31.8%) fair perception, and 220 (59.8%) good perception of AEFI (Figure 2).

**Table 3:** Participants' responses to the perception items on AEFI (*N* = 368).

Perception item	Yes, *n* (%)	No, *n* (%)
PQ1. Do you think there is nothing to worry about AEFI?	245 (66.6)	123 (33.4)
PQ2. Do you think AEFI is a punishment from the gods of the land for receiving a vaccine?	47 (12.8)	321 (87.2)
PQ3. Do you think HCWs should be held responsible for every case of AEFI?	165 (44.8)	203 (55.2)
PQ4. Do you think all forms of AEFI should be managed at home?	157 (42.7)	211 (57.3)
PQ5. If your child has AEFI, would that stop you from receiving other vaccines?	62 (16.8)	306 (83.2)
PQ6. Do you think it is necessary to report all cases of AEFI?	253 (68.8)	115 (31.2)
PQ7. Do you think HCWs will blame you if you report AEFI to them?	151 (41.0)	217 (59.0)
PQ8. Do you think the appropriate place to report AEFI is the nearest patent medicine seller?	76 (20.7)	292 (79.3)
PQ9. Do you think reporting of AEFI will improve the vaccination program?	298 (81.0)	70 (19.0)
PQ10. Do you think reporting AEFI is a waste of time?	86 (23.4)	282 (76.6)

Abbreviations: AEFI = adverse events following immunization, HCW = health care worker, PQ = perception question.

### 3.4 Correlation Between Participants' Knowledge and Perception of AEFI

The mean knowledge and perception scores were 7.6 ± 2.2 and 6.8 ± 2.2 out of 10, respectively. There was a positive, moderate, and statistically significant correlation between knowledge and perception scores (Pearson's *r* = 0.442, *n* = 368, *p *< 0.001), indicating that approximately 20% of the variance in perception scores (*r* ^2 ^= 0.195) was shared with knowledge scores.

### 3.5 Factors Associated With Participants' Knowledge of AEFI (Bivariate Analysis)

On bivariate analysis, age group (*χ* ^2 ^= 22.539, df = 6, *p *< 0.001) and residential status (*χ* ^2 ^= 7.254, df = 2, *p*= 0.03) were significantly associated with the grade of knowledge. Sex, relationship to the vaccinated child, educational status, occupational status, and socioeconomic class were not significantly associated with knowledge (all *p*> 0.05). The underlying frequencies are shown in Table 4.

**Table 4:** Factors associated with participants' knowledge of AEFI (bivariate analysis, *N* = 368).

Variable	Poor (0–3) *n* (%)	Fair (4–6) *n* (%)	Good (7–10) *n* (%)	*χ* ^2^	df	*p* value
Age group (years)						
15–24	0 (0.0)	4 (4.3)	14 (5.5)	22.539	6	< 0.001*
25–34	12 (60.0)	50 (53.2)	138 (54.3)			
35–44	8 (40.0)	35 (37.2)	100 (39.4)			
45–54	0 (0.0)	5 (5.3)	2 (0.8)			
Sex						
Male	0 (0.0)	1 (1.1)	2 (0.8)	0.239	2	0.89
Female	20 (100.0)	93 (98.9)	252 (99.2)			
Residential status						
Within Osogbo	17 (85.0)	72 (76.6)	224 (88.2)	7.254	2	0.03*
Outside Osogbo	3 (15.0)	22 (23.4)	30 (11.8)			
Relationship to the vaccinated child						
Mother	20 (100.0)	93 (98.9)	245 (96.5)	3.438	4	0.49
Father	0 (0.0)	1 (1.1)	2 (0.8)			
Aunt	0 (0.0)	0 (0.0)	7 (2.8)			
Educational status						
Primary	4 (20.0)	14 (14.9)	20 (7.9)	12.141	6	0.06
Secondary	8 (40.0)	49 (52.1)	119 (46.9)			
Tertiary	7 (35.0)	29 (30.9)	113 (44.5)			
Informal	1 (5.0)	2 (2.1)	2 (0.8)			
Occupational status						
Civil servant	4 (20.0)	22 (23.4)	62 (24.4)	4.972	8	0.76
Artisan	2 (10.0)	19 (20.2)	52 (20.5)			
Trader	13 (65.0)	49 (52.1)	130 (51.2)			
Farmer	0 (0.0)	1 (1.1)	0 (0.0)			
Unemployed	1 (5.0)	3 (3.2)	10 (3.9)			
Socioeconomic class						
Low	10 (50.0)	44 (46.8)	94 (37.0)	4.219	4	0.38
Middle	8 (40.0)	34 (36.2)	116 (45.7)			
Upper/high	2 (10.0)	16 (17.0)	44 (17.3)			

*Note:* Percentages are column percentages within each knowledge grade (poor, *n* = 20; fair, *n* = 94; good, *n* = 254).

Abbreviations: *χ* ^2^ = Pearson chi‐square statistic, df = degrees of freedom.

* Statistically significant at *p* < 0.05.

### 3.6 Independent Predictors of Participants' Knowledge of AEFI (Multivariate Analysis)

Age group and residential status were entered into a multinomial logistic regression model with good knowledge as the reference outcome category (Table 5). Caregivers aged 25–34 years were about 70% less likely than those aged 45–54 years to be graded fair rather than good (AOR 0.30, 95% CI 0.089–0.998, *p* = 0.05); in other words, caregivers in this age band were more likely to attain good knowledge. Caregivers residing within Osogbo were about 56% less likely than those residing outside Osogbo to be graded fair rather than good (AOR 0.44, 95% CI 0.225–0.842, *p* = 0.01). Neither age group nor residential status was independently associated with poor rather than good knowledge (all *p* > 0.05). The upper limit of the confidence interval for the 25–34 year age group lies very close to unity, and only two participants aged 45–54 years were graded good, so this particular estimate is imprecise and should be interpreted with caution.

**Table 5:** Independent predictors of participants' knowledge of AEFI (multinomial logistic regression, *N* = 368).

Predictor	AOR	95% CI	*p* value
Fair knowledge versus good knowledge (reference outcome: good knowledge)			
*45–54 years (reference)*	*1.00*	*—*	*—*
15–24 years	2.10	0.412–10.742	0.37
25–34 years	0.30	0.089–0.998	0.05*
35–44 years	0.34	0.100–1.169	0.09
*Outside Osogbo (reference)*	*1.00*	—	—
Within Osogbo	0.44	0.225–0.842	0.01*
Poor knowledge versus good knowledge (reference outcome: good knowledge)			
*45–54 years (reference)*	*1.00*	*—*	*—*
15–24 years	0.89	0.156–5.102	0.90
25–34 years	0.65	0.201–2.069	0.46
35–44 years	1.18	0.356–3.896	0.79
*Outside Osogbo (reference)*	*1.00*	—	—
Within Osogbo	0.76	0.289–1.987	0.57

*Note:* Reference categories are shown first in italics. Model adjusted for age group and residential status.

Abbreviations: AOR = adjusted odds ratio, CI = confidence interval.

* Statistically significant at *p* < 0.05.

### 3.7 Factors Associated With Participants' Perception of AEFI (Bivariate Analysis)

On bivariate analysis, age group (*χ* ^2 ^= 13.005, df = 6, *p* = 0.04), residential status (*χ* ^2 ^= 8.836, df = 2, *p* = 0.01), educational status (*χ* ^2 ^= 31.576, df = 6, *p* < 0.001), occupational status (*χ* ^2 ^= 17.334, df = 8, *p* = 0.03) and socioeconomic class (*χ* ^2 ^= 25.063, df = 4, *p *< 0.001) were significantly associated with the grade of perception of AEFI (Table 6). Sex and relationship to the vaccinated child were not significantly associated with perception (*p* > 0.05).

**Table 6:** Factors associated with participants' perception of AEFI (bivariate analysis, *N* = 368).

Variable	Poor (0–3) *n* (%)	Fair (4–6) *n* (%)	Good (7–10) *n* (%)	*χ* ^2^	df	*p* value
Age group (years)						
15–24	1 (3.2)	3 (2.6)	14 (6.4)	13.005	6	0.04*
25–34	14 (45.2)	68 (58.1)	118 (53.6)			
35–44	16 (51.6)	41 (35.0)	86 (39.1)			
45–54	0 (0.0)	5 (4.3)	2 (0.9)			
Sex						
Male	0 (0.0)	1 (0.9)	2 (0.9)	0.281	2	0.87
Female	31 (100.0)	116 (99.1)	218 (99.1)			
Residential status						
Within Osogbo	25 (80.6)	91 (77.8)	197 (89.5)	8.836	2	0.01*
Outside Osogbo	6 (19.4)	26 (22.2)	23 (10.5)			
Relationship to the vaccinated child						
Mother	31 (100.0)	115 (98.2)	212 (96.4)	2.386	4	0.67
Father	0 (0.0)	1 (0.9)	2 (0.9)			
Aunt	0 (0.0)	1 (0.9)	6 (2.7)			
Educational status						
Primary	4 (12.9)	23 (19.7)	11 (5.0)	31.576	6	< 0.001*
Secondary	21 (67.7)	58 (49.6)	97 (44.1)			
Tertiary	6 (19.4)	34 (29.0)	109 (49.5)			
Informal	0 (0.0)	2 (1.7)	3 (1.4)			
Occupational status						
Civil servant	4 (12.9)	17 (14.5)	67 (30.5)	17.334	8	0.03*
Artisan	8 (25.8)	23 (19.7)	42 (19.1)			
Trader	19 (61.3)	70 (59.8)	103 (46.8)			
Farmer	0 (0.0)	1 (0.9)	0 (0.0)			
Unemployed	0 (0.0)	6 (5.1)	8 (3.6)			
Socioeconomic class						
Low	14 (45.2)	64 (54.7)	70 (31.8)	25.063	4	< 0.001*
Middle	17 (54.8)	40 (34.2)	101 (45.9)			
Upper/high	0 (0.0)	13 (11.1)	49 (22.3)			

*Note:* Percentages are column percentages within each perception grade (poor, *n* = 31; fair, *n* = 117; good, *n* = 220).

Abbreviations: *χ* ^2^ = Pearson chi‐square statistic, df = degrees of freedom.

* Statistically significant at *p* < 0.05.

### 3.8 Independent Predictors of Participants' Perception of AEFI (Multivariate Analysis)

The five variables significant on bivariate analysis (age group, residential status, educational status, occupational status, and socioeconomic class) were entered into a multinomial logistic regression model with good perception as the reference outcome category (Table 7). After adjustment, only age group and residential status remained independently associated with perception; educational status, occupational status, and socioeconomic class were no longer significant (all *p* > 0.05), suggesting that their bivariate associations were largely mediated by, or confounded with, age and place of residence. Caregivers aged 25–34 years had higher odds of poor rather than good perception compared with those aged 45–54 years (AOR 1.46, 95% CI 1.12–1.91, *p *< 0.001), and caregivers residing within Osogbo had lower odds of fair rather than good perception compared with those residing outside Osogbo (AOR 0.44, 95% CI 0.224–0.852, *p* = 0.02).

**Table 7:** Independent predictors of participants' perception of AEFI (multinomial logistic regression, *N* = 368).

Predictor	AOR	95% CI	*p* value
Age group (years)			
*Good perception (reference category)*	1.00		
Poor perception vs. good perception			
15–24	1.32	0.89–1.96	0.07
25–34	1.46	1.12–1.91	< 0.001*
35–44	0.98	0.71–1.35	0.89
45–54 (reference)	1.00		
Fair perception vs. good perception			
15–24	0.76	0.20–2.84	0.68
25–34	1.10	0.40–3.02	0.85
35–44	0.87	0.31–2.43	0.79
45–54 (reference)	1.00		
Residential status			
*Good perception (reference category)*	1.00		
Fair perception vs. good perception			
Within Osogbo	0.44	0.224–0.852	0.02*
Outside Osogbo (reference)	1.00		
Poor perception vs. good perception			
Within Osogbo	0.76	0.321–1.802	0.53
Outside Osogbo (reference)	1.00		

*Note:* Reference categories are shown first in italics. Model adjusted for age group, residential status, educational status, occupational status, and socioeconomic class; the last three were not independently significant, and their estimates are omitted for clarity.

Abbreviations: AOR = adjusted odds ratio, CI = confidence interval.

* Statistically significant at *p* < 0.05.

## 4 Discussion

This study assessed the knowledge and perception of caregivers regarding adverse events following immunization and the factors associated with them, and examined the correlation between the two. To the best of our knowledge, it is the first study from Osun State to measure caregiver knowledge and perception of AEFI in the same population, to quantify the relationship between them, and to identify independent correlates after adjustment.

### 4.1 Knowledge of AEFI and Its Correlates

The proportion of participants with good knowledge of AEFI in this study (69.0%) was higher than that reported by Afolaranmi et al. [6] in Jos, North Central Nigeria (31.5%), but lower than the 78.9% adequate knowledge reported by Issa‐Onilu et al. [7] in Ilorin. These differences may reflect variation in geographical location, study setting (hospital‐only vs. mixed community and facility recruitment), scoring thresholds and sociocultural characteristics. More important than the headline figure, however, is where the knowledge fails: only 216 participants (58.7%) knew that an AEFI may occur without a direct causal link to the vaccine. This is the single item most closely tied to how caregivers behave after an event, because a caregiver who attributes every post‐vaccination illness to the vaccine is the caregiver most likely to withhold the next dose. Aggregate “good knowledge” therefore overstates the adequacy of caregiver understanding, and programs should track item‐level rather than composite performance.

Regarding correlates of knowledge, age group and residential status were significant. Caregivers aged 25–34 years had lower odds of fair rather than good knowledge than those aged 45–54 years, suggesting better knowledge among younger caregivers. This may be because younger caregivers represent the most mobile demographic, with greater exposure to health information through digital media and greater eagerness to acquire it. Studies have shown that individuals aged 18–40 years demonstrate appreciable increases in the quantity and quality of knowledge of a subject [11]. In contrast, age was not associated with knowledge of AEFI in a study conducted in the United Arab Emirates by John et al. [12]. Residing within Osogbo, an urban center, was likewise associated with better knowledge, plausibly because of greater access to health information, health facilities, and formal and informal health communication; residential location has previously been shown to influence knowledge acquisition [13].

### 4.2 Perception of AEFI and Its Correlates

The proportion of participants with a good perception of AEFI in this study was similar to that reported among vaccination workers by Lv et al. in Zhejiang province, China [8], and to that reported in Ghana [5]. This similarity may be attributable to comparable knowledge levels and sociodemographic characteristics of the participants.

On bivariate analysis, age group, residential status, educational status, occupational status, and socioeconomic class were all associated with perception, but only age group and residential status remained significant after adjustment. This attenuation is itself informative: education, occupation, and socioeconomic class are strongly interrelated in this population, and their apparent bivariate effects appear to operate largely through the same underlying pathway of access to information. Progressive increase in caregiver age may shape perception through the knowledge and experience of immunization accumulated over successive children [14], while higher educational attainment is consistently linked with more favorable health outcomes and better health literacy. These observations support the view that perception is dynamic and modifiable rather than fixed [15].

Socioeconomic position has previously been shown to influence perception of AEFI and vaccine hesitancy, with suboptimal health outcome indices correlating with lower economic and social resources [16, 17]. Similarly, occupational status has been associated with perception of AEFI in other studies [8, 18, 19]. Highly skilled occupations plausibly influence perception through higher educational attainment, higher health literacy, and greater exposure to formal health information, whereas caregivers in unskilled occupations are less likely to have had extended formal education, which limits their understanding of AEFI and shapes their perception accordingly.

### 4.3 Relationship Between Knowledge and Perception

Knowledge and perception scores were moderately and positively correlated (*r* = 0.442), consistent with the position that advancement in knowledge is a catalyst for change in perception [20]. The practical implication is twofold. On the one hand, interventions that improve knowledge of AEFI can reasonably be expected to shift perception and, in turn, reporting behavior and immunization uptake. On the other hand, the shared variance was only about 20%, so knowledge alone is not sufficient; trust in health workers, prior personal experience, and community narratives evidently account for much of the remainder. This is consistent with the finding that 151 caregivers (41.0%) believed health workers would blame them for reporting an AEFI, a belief that no amount of factual instruction about AEFI will by itself dislodge.

### 4.4 Implications for Practice and Policy

Approximately 7 of every 10 participants had good knowledge, and 6 of every 10 had good perception of AEFI. Clear gaps therefore remain, and the findings point to several concrete and targeted actions rather than to generic calls for health education.

First, pre‐vaccination counseling at the point of service should explicitly distinguish coincidental events from vaccine product‐related reactions, since this was the weakest knowledge item. Second, because caregivers with primary education and those in the lower socioeconomic class had the poorest perception scores, educational materials should be produced in Yoruba as well as English and should use pictorial and audio formats rather than text‐heavy leaflets. Third, because caregivers residing outside Osogbo consistently performed less well, outreach and mobile immunization sessions serving peri‐urban and rural catchment areas should carry the same counseling package as urban facilities rather than a reduced one. Fourth, health workers should be trained to receive AEFI reports without blame, and this should be reinforced by supportive supervision, given the substantial proportion of caregivers who anticipated being blamed. Fifth, social media platforms and trusted community influencers, including religious and traditional leaders, can be enlisted to correct misinformation and to reach the younger caregivers who constitute the majority of this population.

These actions align with the Sustainable Development Goals, the set of 17 interlinked global goals adopted by all United Nations member states in 2015 as part of the 2030 Agenda for Sustainable Development, which provide the framework against which national health and education investments are prioritized and monitored [21]. Goal 3 (good health and well‐being) and Goal 4 (quality education) are directly relevant here: reducing preventable under‐five deaths, which is target 3.2, depends on sustained immunization coverage, and sustained coverage depends in turn on caregivers who understand and correctly interpret adverse events following immunization.

### 4.5 Strengths and Limitations

The main strengths of this study are that it recruited from the immunization units of the three major hospitals in Osogbo across tertiary, secondary public, and mission facility levels, that participants were selected by proportionate systematic random sampling rather than by convenience, and that knowledge and perception were assessed in the same participants using a pretested instrument with acceptable internal consistency.

Several limitations should be borne in mind. The most important is inherent in the study design: data were collected cross‐sectionally, at a single point in time, so temporal sequence cannot be established and no causal inference can be drawn about the relationships reported here [22]. Participants' responses may also have been influenced by contemporaneous events, media coverage, or facility‐level experiences that were not measured in this study, and residual confounding by unmeasured factors such as previous personal experience of an AEFI, health worker communication practices, and religious beliefs cannot be excluded. Also, the data were self‐reported and therefore subject to social desirability and recall bias, mitigated but not eliminated by assurance of confidentiality, anonymity of questionnaires, and emphasis on candid responses.

## 5 Conclusion

Knowledge and perception of AEFI among hospital‐attending caregivers in Osogbo were largely good, but important and specific gaps remain, most notably the widespread belief that every event following immunization is caused by the vaccine. Younger caregivers and urban residents were independently more likely to have good knowledge and perception, while lower educational attainment, lower occupational status, and lower socioeconomic class were associated with poorer perception at the bivariate level. Interventions that combine point‐of‐service counseling on causality, vernacular and pictorial materials for caregivers with limited formal education, equivalent counseling in peri‐urban outreach sessions, and blame‐free handling of AEFI reports by health workers, are likely to strengthen AEFI literacy and thereby reduce vaccine hesitancy and immunization dropout.

## Author Contributions

**Funso Abidemi Olagunju:** conceptualization, supervision, project administration, writing – review and editing, methodology, writing – original draft. **Samuel Olorunyomi Oninla:** conceptualization, methodology, supervision, writing – review and editing. **Sunday Charles Adeyemo:** conceptualization, methodology, project administration, writing – review and editing, writing – original draft. **Abimbola Ololade Odeyemi:** data curation, investigation, writing – review and editing. **Kehinde Awodele:** investigation, writing – review and editing, validation. **Efeturi Agelebe:** formal analysis, writing – review and editing, data curation. **Abisola Oluwatoyin Omoboyeje:** data curation, writing – review and editing. **Muritala Adewale Ibrahim:** visualization, writing – review and editing, data curation. **Ayodeji Oluwaseun Ogungbemi:** software, writing – review and editing. **Ayodele Raphael Ajayi:** formal analysis, writing – review and editing. **Eniola Dorcas Olabode:** writing – original draft, writing – review and editing, formal analysis. **Oladunni Opeyemi:** data curation, writing – review and editing, investigation. **Olusola Adetunji Oyedeji:** data curation, writing – review and editing.

## Funding

This research received no specific grant from any funding agency in the public, commercial or not‐for‐profit sectors. The study was funded entirely by the authors. No funding source or financial relationship had any involvement in the study design; in the collection, analysis or interpretation of data; in the writing of the report; or in the decision to submit the report for publication.

## Ethics Statement

This study was conducted in accordance with the ethical principles outlined in the Declaration of Helsinki. Ethical approval was obtained from the Osun State Health Research Ethics Committee (protocol number OSHREC/PRS/569T/1279).

## Consent

Written informed consent was obtained from all participants prior to their participation in the study. Participation was voluntary, and participants were free to withdraw at any time without consequence.

## Conflicts of Interest

The authors declare no conflicts of interest.

## Transparency Statement

Sunday Charles Adeyemo, the manuscript guarantor, affirms that this manuscript is an honest, accurate and transparent account of the study being reported; that no important aspects of the study have been omitted; and that any discrepancies from the study as planned (and, if relevant, registered) have been explained.

## Data Availability

The data that support the findings of this study are not publicly available because the conditions of the ethical approval and the consent obtained from participants do not permit open deposition of the individual‐level questionnaire responses. The fully anonymised dataset and the study questionnaire are available from the corresponding author (Sunday Charles Adeyemo, charlespatho@gmail.com) on reasonable request, subject to the approval of the Osun State Health Research Ethics Committee and the completion of a data‐sharing agreement. All other data supporting the findings of this study are available within the article.
